# Internet-Administered Cognitive Behavioral Therapy for Common Mental Health Difficulties in Parents of Children Treated for Cancer: Intervention Development and Description Study

**DOI:** 10.2196/22709

**Published:** 2021-07-22

**Authors:** Joanne Woodford, Paul Farrand, Josefin Hagström, Li Hedenmalm, Louise von Essen

**Affiliations:** 1 Uppsala University Healthcare Sciences and e-Health Department of Women’s and Children’s Health Uppsala Sweden; 2 Clinical Education, Development, and Research (CEDAR) Psychology, College of Life and Environmental Sciences University of Exeter Exeter United Kingdom

**Keywords:** parents, eMental health, internet-administered cognitive behavioral therapy, ICBT, TIDieR, CBT self-help, low-intensity CBT, mobile phone

## Abstract

**Background:**

Following the end of a child’s treatment for cancer, parents may report psychological distress. However, there is a lack of evidence-based interventions that are tailored to the population, and psychological support needs are commonly unmet. An internet-administered low-intensity cognitive behavioral therapy (LICBT)–based intervention (EJDeR [internetbaserad självhjälp för föräldrar till barn som avslutat en behandling mot cancer]) may provide a solution.

**Objective:**

The first objective is to provide an overview of a multimethod approach that was used to inform the development of the EJDeR intervention. The second objective is to provide a detailed description of the EJDeR intervention in accordance with the Template for Intervention Description and Replication (TIDieR) checklist.

**Methods:**

EJDeR was developed through a multimethod approach, which included the use of existing evidence, the conceptualization of distress, participatory action research, a cross-sectional survey, and professional and public involvement. Depending on the main presenting difficulty identified during assessment, LICBT behavioral activation or worry management treatment protocols are adopted for the treatment of depression or generalized anxiety disorder when experienced individually or when comorbid. EJDeR is delivered via the Uppsala University Psychosocial Care Programme (U-CARE) portal, a web-based platform that is designed to deliver internet-administered LICBT interventions and includes secure videoconferencing. To guide parents in the use of EJDeR, weekly written messages via the portal are provided by e-therapists comprising final year psychology program students with training in cognitive behavioral therapy.

**Results:**

An overview of the development process and a description of EJDeR, which was informed by the TIDieR checklist, are presented. Adaptations that were made in response to public involvement are highlighted.

**Conclusions:**

EJDeR represents a novel, guided, internet-administered LICBT intervention for supporting parents of children treated for cancer. Adopting the TIDieR checklist offers the potential to enhance fidelity to the intervention protocol and facilitate later implementation. The intervention is currently being tested in a feasibility study (the ENGAGE study).

**International Registered Report Identifier (IRRID):**

RR2-10.1136/bmjopen-2018-023708

## Introduction

### Background

Each year, approximately 300,000 children and young people (aged 0-19 years) are diagnosed with cancer worldwide [1]. Despite significant treatment advances resulting in a 5-year survival rate of 81.2% across Northern Europe [2], childhood cancer remains a leading cause of death [3] and disease burden [4] among children worldwide. As parents are the primary source of support for children with cancer, they are faced with significant negative psychological [5-10] and socioeconomic [11-15] impacts, along with increased caregiving burden [16] and poor health-related quality of life [17].

Compared with population controls, parents of children treated for cancer report a higher prevalence of mental health difficulties, including depression, anxiety, and posttraumatic stress symptoms [6-10]. Despite the prevalence of mental health difficulties, parents report a number of significant barriers to accessing psychological treatment to meet their needs [18-20]. These barriers occur at the individual level: lack of time, putting the needs of their child first, and guilt [21,22]; provider level: lack of knowledge of mental health difficulties and willingness to diagnose and treat mental health problems; and systemic level: limited availability of trained and qualified health care providers [23-25].

Innovative strategies to address barriers and improve access to evidence-based psychological interventions are being implemented worldwide [26]. One such innovation is the Improving Access to Psychological Therapies (IAPT) program in England [27,28], which is now also being piloted in countries including Australia [29] and Norway [30]. The IAPT program was established in recognition that improving access to evidence-based psychological therapies required a fundamental transformation of mental health service delivery. This transformation was achieved through the delivery of psychological treatments within a stepped care service delivery model [31]. One important feature of the stepped care model is that the least restrictive evidence-based treatment available that is likely to result in a significant health gain is provided initially [32,33]. For example, lower demands placed on patients in terms of cost and personal inconvenience [32,33]. At step 2, low-intensity cognitive behavioral therapy (LICBT) is provided by a psychological practitioner workforce trained in competencies to support patients to engage in LICBT interventions [34]. At step 3, high-intensity cognitive behavioral therapy (HICBT) is delivered to patients, primarily face-to-face, by traditional psychological therapists.

LICBT interventions are delivered through a range of cognitive behavioral therapy (CBT) self-help interventions, including print-based formats or e-mental health (eg, internet administered and smartphone apps) formats [35]. Using LICBT interventions to deliver specific CBT techniques enables treatment to be provided with shorter session times while ensuring that patients receive a similar dose of therapy to that delivered by HICBT therapists [34]. With HICBT, evidence-based treatment protocols specify the delivery of several CBT techniques as part of a multistrand approach, such as cognitive therapy for depression [36]. With LICBT, a single-strand approach is adopted, in which a clinical decision is made to adopt a single evidence-based CBT technique for the treatment of a specific, common mental health difficulty [34]. Given the evidence base highlighting larger effect sizes associated with guided LICBT versus those associated with self-administered LICBT [37,38], interventions are supported by a psychological practitioner workforce [34].

The evidence base for LICBT has been demonstrated in over 30 systematic reviews and 50 controlled trials [39]. Controlled trials of guided internet-administered LICBT interventions versus face-to-face psychological therapies have been demonstrated to produce equivalent overall effects [40], and acceptability has been demonstrated in usual care settings [41]. In addition to placing fewer demands on parents of children treated for cancer, guided internet-administered LICBT may represent a solution to address individual-and provider-level barriers to access [42-44]. An existing internet-administered CBT intervention for parents of children treated for cancer has been found to be acceptable and feasible [45]. However, this was an HICBT intervention, delivered in real time by a qualified psychologist using a group treatment format. To the best of our knowledge, there is no guided internet-administered LICBT intervention for parents of children treated for cancer.

### Objectives

The objectives are twofold. The first objective is to provide an overview of the multimethod approach informing the development of a guided internet-administered LICBT intervention for parents of children treated for cancer (EJDeR [internetbaserad självhjälp för föräldrar till barn som avslutat en behandling mot cancer]), following phase I (development) of the Medical Research Council complex interventions framework [46]. The second objective is to provide a detailed description of the EJDeR intervention in accordance with the Template for Intervention Description and Replication (TIDieR) checklist [47] to overcome criticisms concerning poor and incomplete reporting of complex nonpharmacological interventions [48].

## Methods

### Overview

**Figure 1:**
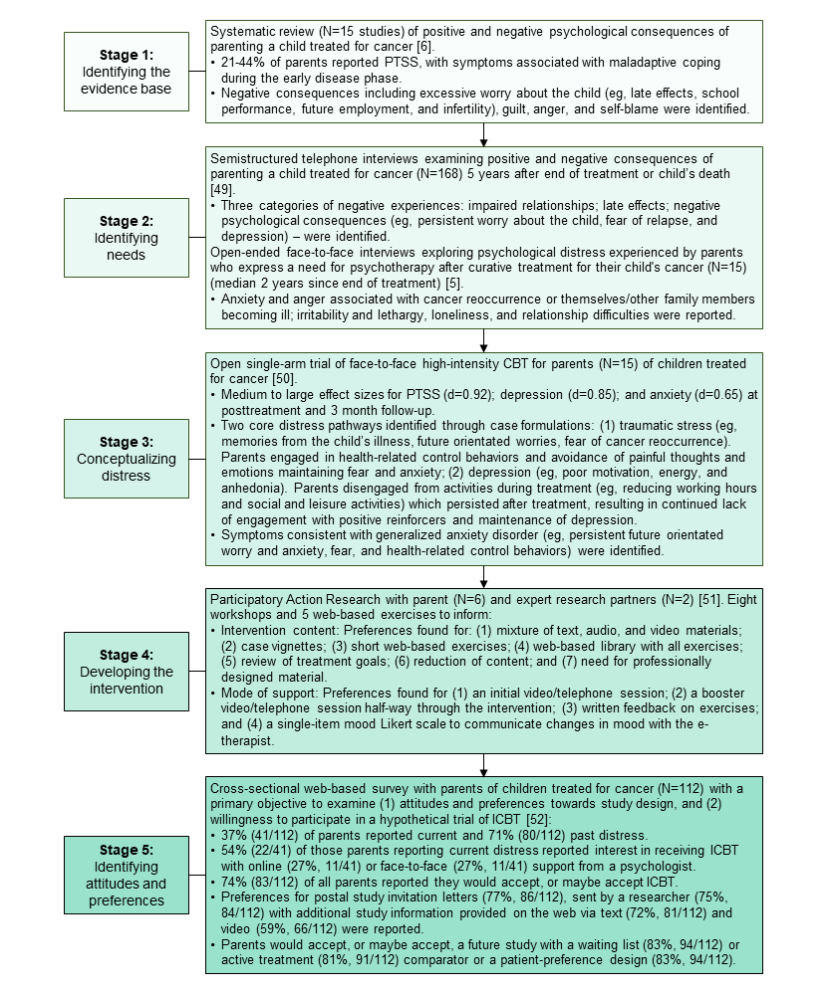
Previous research informing the development of EJDeR. CBT: cognitive behavioral therapy; EJDeR: internetbaserad självhjälp för föräldrar till barn som avslutat en behandling mot cancer; ICBT: internet-administered cognitive behavioral therapy; PTSS: posttraumatic stress symptoms.

**Figure 2:**
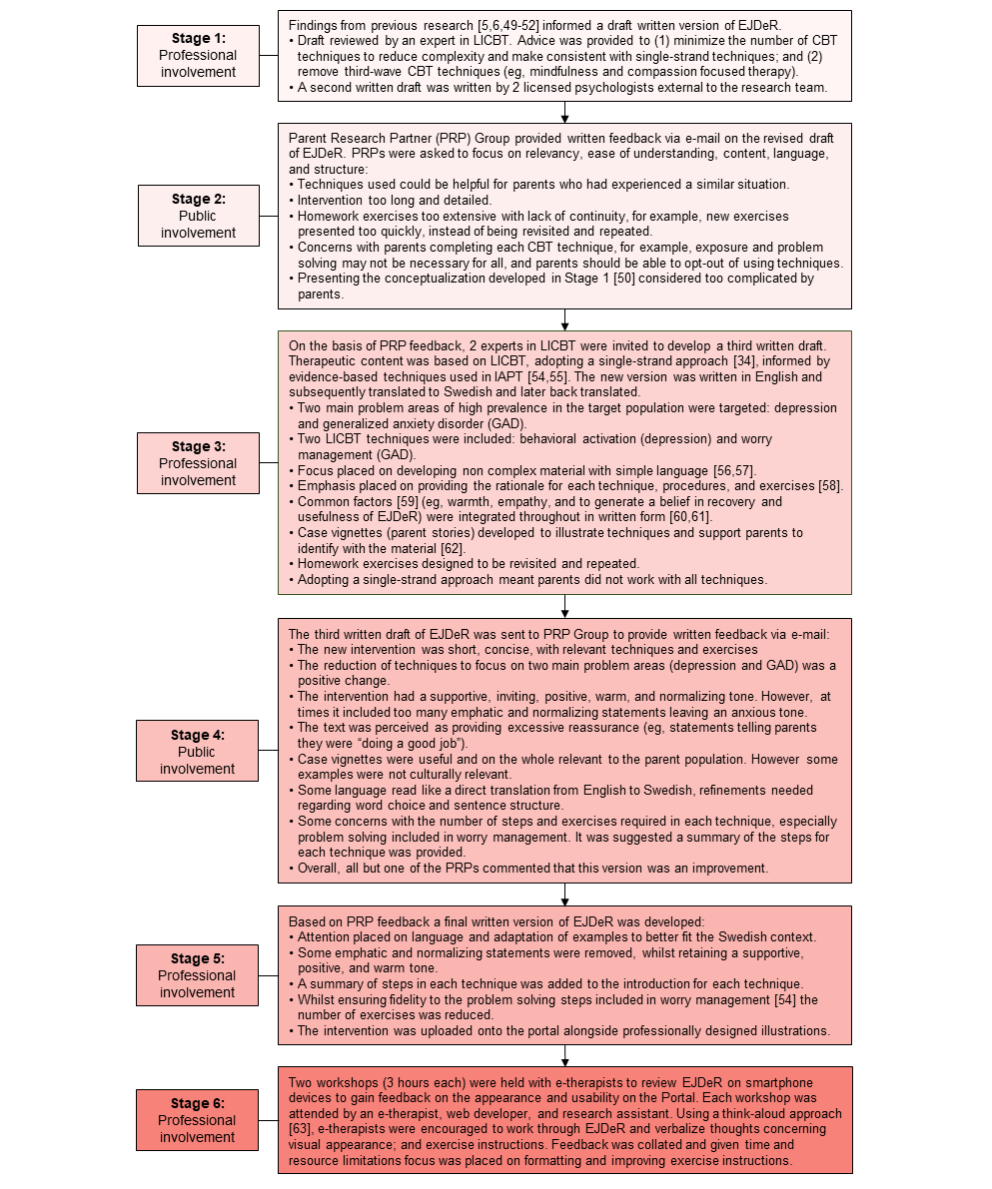
Public and professional involvement. CBT: cognitive behavioral therapy; EJDeR: internetbaserad självhjälp för föräldrar till barn som avslutat en behandling mot cancer; GAD: generalized anxiety disorder; IAPT: Improving Access to Psychological Therapies; LICBT: low-intensity cognitive behavioral therapy; PRP: parent research partner.

Mixed methods, including a systematic review [6], interview studies [5,49], a single-arm trial [50], participatory action research [51], and a cross-sectional web-based survey [52] informed the initial development of EJDeR (Figure 1). Subsequently, public [53] and professional involvement was adopted to improve the quality, relevance, and acceptability of the intervention. The parent research partner (PRP) group consisted of 2 mothers and 2 fathers of a child treated for cancer who were aged between 45 and 54 years and recruited via word of mouth. Professional involvement included collaboration with a multidisciplinary team of licensed clinical psychologists, e-therapists, pediatric oncologists, and web developers (Figure 2). We included 10 publications [54-63] in Figure 2.

### Ethics

Ethical approval for studies informing the EJDeR intervention development process was granted by the regional ethical review board in Uppsala (DNR: 2012/440; DNR: 2015/426; DNR: 2017/527) and the Swedish Ethical Review Authority (DNR: 2019-03083).

## Results

### Overview of the EJDeR Intervention

EJDeR is a guided internet-administered LICBT intervention for parents 3 months to 5 years following their child ending treatment for cancer. For parents, the end of treatment is a period of psychological vulnerability [6], and a subgroup reports long-term psychological distress after the end of treatment [10]. EJDeR is delivered on the Uppsala University Psychosocial Care Programme (U-CARE) portal (hereafter referred to as the portal), an in-house platform designed to deliver CBT interventions and support data collection [64,65]. EJDeR is intended to be delivered over 12 weeks and consists of 4 modules: (1) introduction and psychoeducation, (2) behavioral activation (BA), (3) worry management, and (4) relapse prevention. First, parents attend an initial assessment via videoconferencing or telephone interviews with an e-therapist. Consistent with an LICBT single-strand approach, a decision is made during the initial assessment to adopt BA to target depression or worry management for generalized anxiety disorder (GAD). Thereafter, e-therapists provide weekly written messages via the portal to guide parents to use the relevant module. Parents also receive a midintervention booster session with their e-therapist via videoconferencing or telephone. On occasions where difficulties remain after completion of BA or worry management, a collaborative decision may be reached to progress to the other LICBT technique. A detailed description of EJDeR is provided below in accordance with the items included in the TIDieR checklist [47].

### TIDieR Checklist Item 1: Brief Name of the Intervention

The intervention was named EJDeR, which is a Swedish acronym for internetbaserat självhjälpsprogram för föräldrar till barn som avslutat en behandling mot cancer.

### TIDieR Checklist Item 2: Rationale, Theory, or Goal of the Intervention

#### Overview

Theory related to the CBT model informing the development and maintenance of psychological distress was applied to understand the etiology and maintenance of distress in parents of children treated for cancer [50]. On the basis of the resulting conceptualization of distress in the population [50], depression and traumatic stress were proposed as the main psychological difficulties likely to arise in the population. Symptoms consistent with GAD (eg, persistent future-orientated worry and anxiety, fear, and health-related control behaviors) were also identified [50]. Given that depression and GAD are recommended for treatment with LICBT, EJDeR was developed to target depression and GAD, rather than posttraumatic stress disorder (PTSD) given the lack of evidence base for LICBT for PTSD [66]. Consistent with LICBT, EJDeR comprises two separate single-strand LICBT techniques: BA [67-69] and worry management [54,70,71] to target depression and GAD, respectively. EJDeR is not designed to support parents with a diagnosis of severe or enduring mental health difficulties or parents who are suicidal or have a history of persistent self-harm.

#### BA for Depression

To prioritize their child’s cancer treatment, parents of children receiving cancer treatment commonly disengage from activities that make up a normal life routine, such as decreased engagement in work, social activities, and everyday household tasks [5,49,50]. At the time of the child’s illness, prioritizing their child’s cancer treatment can be helpful for parents in the short term to manage the difficult situation of being a parent to a child with cancer. However, even after treatment has ended, some parents continue to disengage from these activities. This can arise as a consequence of negative reinforcement, whereby continuing to focus on their child’s needs at the expense of their own and not re-engaging with previously undertaken activities can provide relief. However, failing to re-engage with previous activities, in particular those found pleasant, reduces opportunities for positive reinforcement, whereas engagement in unnecessary activities associated with their child’s treatment is maintained through negative reinforcement [67,69,72,73]. To break this maintenance cycle, EJDeR adopts an LICBT BA technique [69] theoretically informed by Hopko et al [74] to overcome sources of negative reinforcement and increase engagement with pleasurable activities in a structured and graded way [67-69].

#### Worry Management for GAD

Worry in parents of children treated for cancer is commonly related to the child’s disease. To help avoid potential problems during cancer treatment, or to avoid thinking about the outcome of future threats, parents may engage in worry behavior in an attempt to problem solve current difficulties and avoid future threats [75,76]. When worry is related to a practical problem and results in successful problem solving, it can be highly productive; for example, ensuring the child avoids situations that increase the risk of exposure to infectious diseases [50]. However, worry can be unhelpful when hypothetical; therefore, solutions cannot be generated. For example, concerns related to future cancer reoccurrence in their child or sickness in themselves or family members without any reason [5]. On such occasions, worry may be used as a form of cognitive avoidance to reduce distress and discomfort associated with uncertainty [77,78]. When successful in reducing distress and discomfort, the use of worry behaviors becomes negatively reinforced, helping to manage an intolerance of uncertainty in the long term [77,78]. This intolerance of uncertainty is a core feature of GAD and is common among parents of children treated for cancer [79].

#### Behavior Change Models

To influence the degree to which patients are able to engage with the EJDeR intervention, behavior change theory [80] is integrated to supplement specific factors associated with single-strand LICBT techniques. For example, Self-Determination Theory [81] has been adopted to enhance autonomy, competence, and relatedness. A sense of autonomy is enhanced by providing a clear rationale for each LICBT technique. Clear instructions and guidance on how to complete exercises and guidance and feedback provided by an e-therapist foster competence. A sense of relatedness is established by directing significant attention to the language adopted throughout the intervention, such as the provision of empathy, normalization of common difficulties, and encouraging active engagement.

To complement Self-Determination Theory, the selection, optimization, and compensation (SOC) model [82-84] is embedded to support parents in re-engaging with activities that were given up while supporting their child through cancer treatment or address worry by problem solving practical difficulties faced during treatment. The SOC model has been demonstrated to be a successful strategy for managing the multiple goals associated with different life domains (eg, work, family, and leisure) in middle adulthood [85,86] that may be experienced by parents of children treated for cancer. Within BA, the SOC model is used to support parents in replacing activities that were necessary to stop by selecting other activities that are more achievable and remain of importance and value. The SOC model can help support problem solving by adapting activities in the event of experiencing changes in resources (optimization; eg, lack of time and finance) and identifying ways of achieving the activity in light of changes (compensation; eg, finding time and asking for support). Applying the SOC model enables parents to maximize desirable gains, goals, and outcomes while minimizing undesirable losses, goals, and outcomes [82-84].

### TIDieR Checklist Item 3: Physical or Informational Materials Used in the Intervention Delivery or Training

#### Intervention Delivery

**Figure 3:**
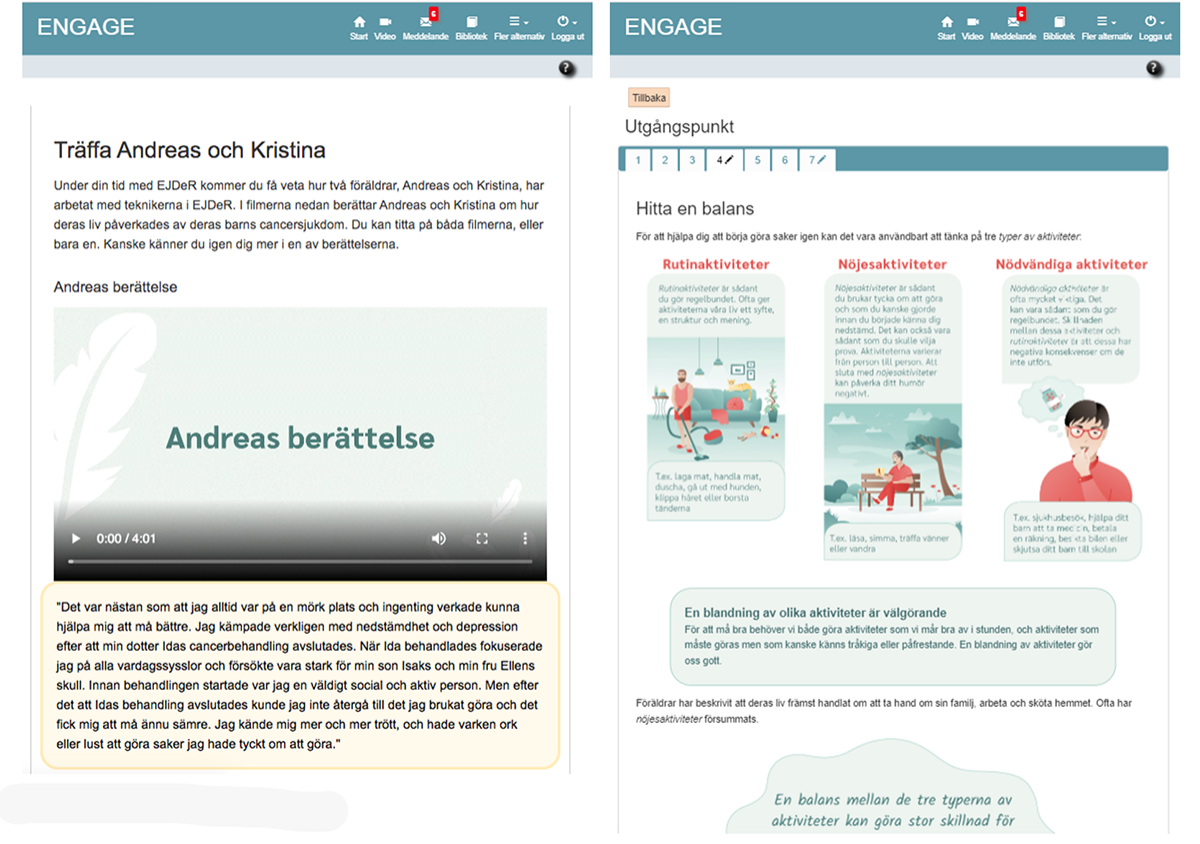
Sample screenshots of EJDeR. EJDeR: internetbaserad självhjälp för föräldrar till barn som avslutat en behandling mot cancer.

EJDeR is delivered on the portal and includes text, illustrations, film, audio files, and a frequently asked questions section. The About Us section presents photos and a brief biography of the EJDeR authors to verify author credibility, previously shown to be important when providing remote treatment [87]. Technical help texts are available throughout EJDeR to support parents to use all functions. To visually present how EJDeR appears to parents, sample screenshots from the intervention can be seen in Figure 3.

**Figure 4:**
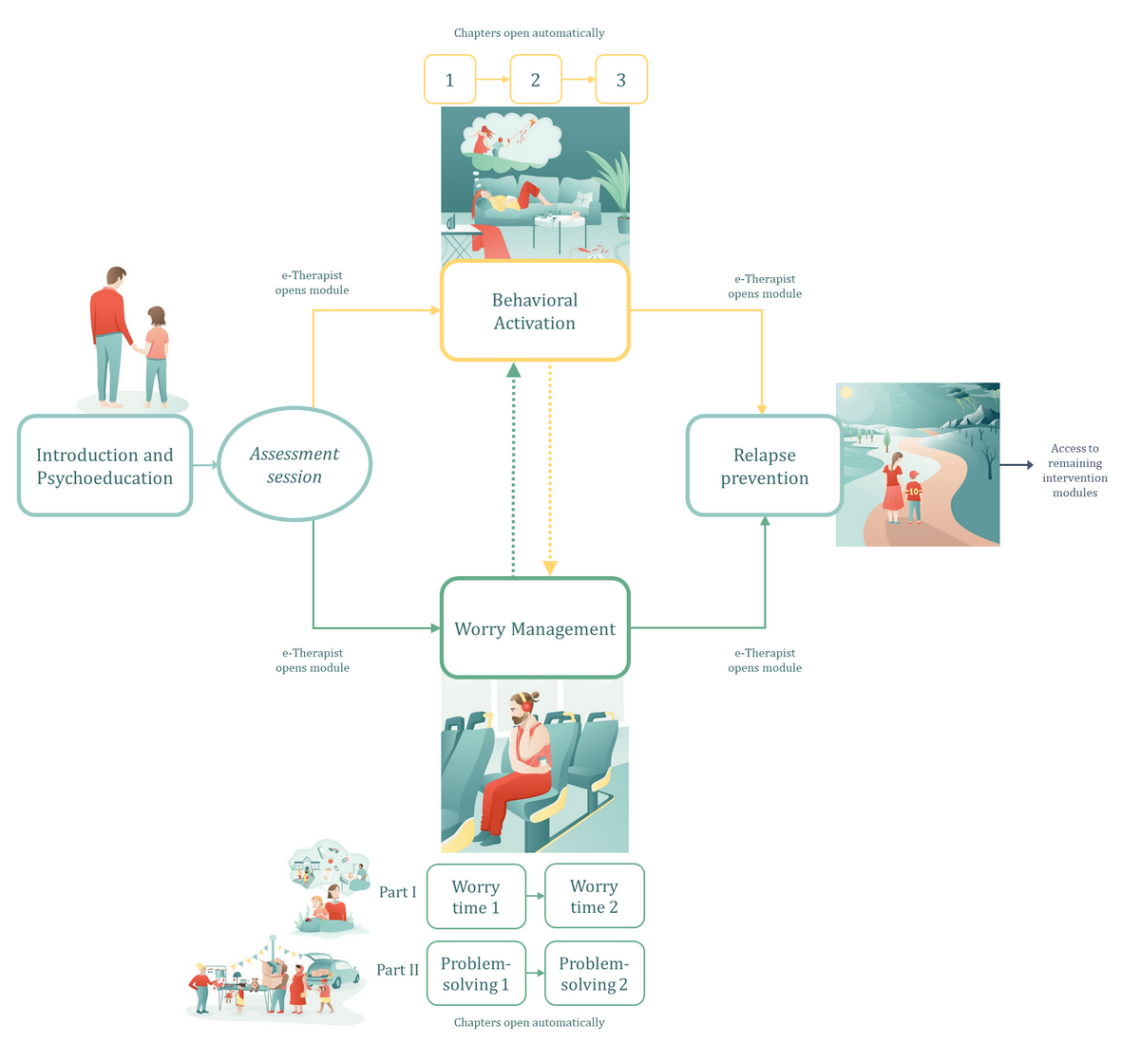
An overview of the structure of EJDeR. EJDeR: internetbaserad självhjälp för föräldrar till barn som avslutat en behandling mot cancer.

Parents initially complete the introduction and psychoeducation module, and after the initial assessment session, e-therapists provide access to the module containing the LICBT technique best suited to their main presenting difficulty (BA or worry management). After completion of BA or worry management, a collaborative decision between the e-therapist and parent may be reached to progress to the other LICBT technique; however, parents only work with a single LICBT technique at a time. A detailed description of the module content is found in TIDieR item 4, and an overview of the structure of EJDeR is shown in Figure 4.

**Figure 5:**
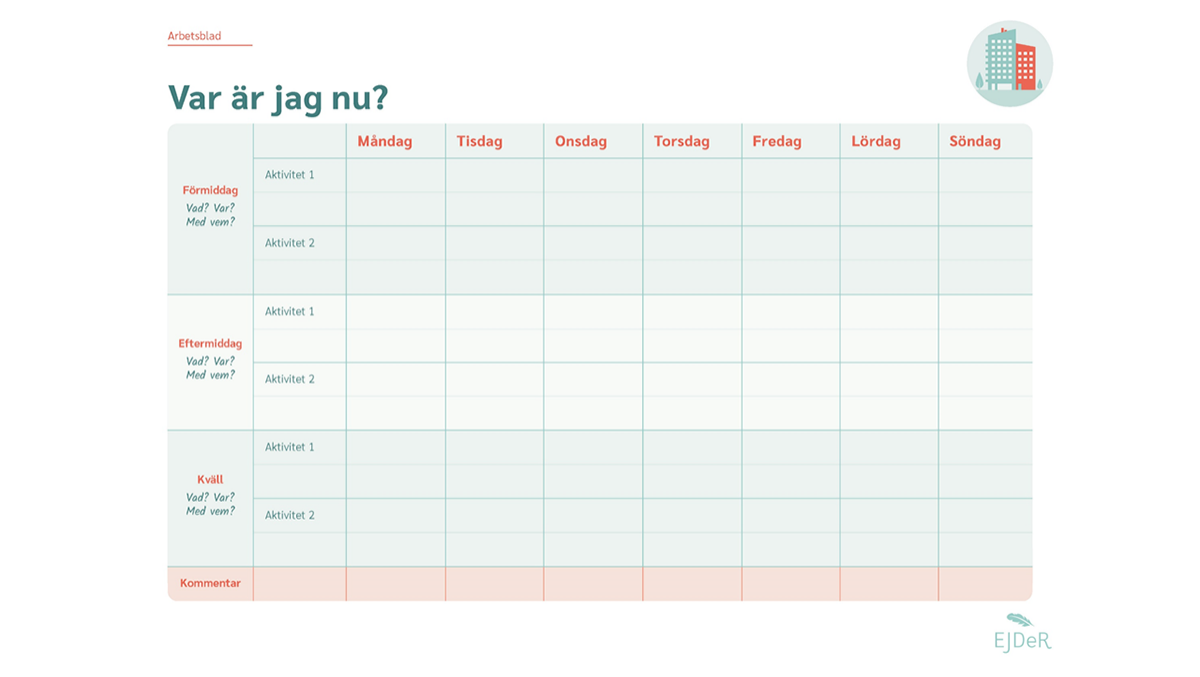
Sample exercise worksheet from EJDeR. EJDeR: internetbaserad självhjälp för föräldrar till barn som avslutat en behandling mot cancer.

Consistent with the LICBT approach, participant engagement with the techniques is facilitated through in-module exercises and weekly homework exercises completed on the portal and submitted to the e-therapist (see Figure 5 for an example). To provide choice, homework exercises can also be printed and completed offline, and parents subsequently complete a weekly homework review on exercise on the portal. Parents can access copies of all weekly homework exercises and audio files in a web-based library in the portal.

#### Intervention Training

e-Therapists are provided with a portal handbook, with instructions on how to use EJDeR and training videos on the delivery of the BA and worry management techniques. e-Therapists review parent progress through the modules and any completed in-module exercises and homework exercises on the portal.

### TIDieR Checklist Item 4: Procedures, Activities, and Processes Used in the Intervention

#### Module: Introduction and Psychoeducation

Parents are provided with a brief introduction of how to use EJDeR. Psychoeducation about psychological distress in the context of being a parent of a child treated for cancer is also provided. Parents are introduced to two case vignettes that are used throughout EJDeR based on the Five Areas CBT model [88,89] to facilitate an understanding of the CBT rationale. To enhance engagement, case vignettes were informed by our previous research [5,51]. Parents (1) complete their own Five Areas CBT model; (2) identify areas of importance and value in their life; and (3) set three goals that are specific, positive, and realistically achievable. Parents are presented with the two case vignettes briefly outlining the techniques parents will work with during EJDeR.

Alongside completion of this module, parents take part in an initial assessment session with an e-therapist (see *TIDieR checklist Item 6*) to determine the parent’s main presenting difficulty. The e-therapist provides access to the BA module for parents experiencing depression and the worry management module for parents experiencing GAD.

#### Module: BA

The full clinical protocol for BA has been described elsewhere [67-69]. Activities that make up a normal life routine are categorized into three types: (1) routine (providing life structure and typically repeated during the week, such as housework and cooking); (2) pleasurable activities that provide a sense of pleasure or enjoyment that are determined by the parent; and (3) necessary activities that are recognized as having the potential for serious negative consequences if not done (eg, attending hospital appointments, taking medication, or paying a bill). Parents are gradually supported to re-engage with activities they have stopped, aiming to re-establish a balance of routine and pleasurable activities, and where required, include necessary activities. The clinical protocol includes four main steps (identifying current activities, identifying stopped activities, organizing activities, and planning activities). As an adaptation, an additional step entitled Prioritizing Activities was added, recognizing that parents commonly experience difficulties trying to balance their home, work, and family life after cancer treatment has ended [5]. Parents may need to reprioritize routine activities to gain opportunities to re-engage with neglected pleasurable activities. A case vignette is used to guide parents through BA, including examples of completed exercises and occasions where setbacks are experienced, and to provide guidance and feedback on the use of BA [60,61]. Parents are encouraged to work with BA, with the exact number of weeks required decided collaboratively between the parent and e-therapist.

#### Module: Worry Management

The clinical protocol for worry management has been described elsewhere [54,70,71]. Parents capture worries over a week in a worry diary and categorize worries into two types: (1) practical (eg, important and can be solved) and (2) hypothetical (eg, important but have no way of being solved, such as worries relating to past events, things that might happen in the future, or things that cannot be controlled). Parents review the types of worries they have captured and determine whether a particular type (eg, practical or hypothetical) has a greater impact and is more distressing. Parents are encouraged to use problem solving for practical worries and worry time for hypothetical worries. A case vignette is also used to guide parents through worry management. Parents continue to work with worry management, with the number of weeks decided collaboratively between the parent and the e-therapist. Parents may work with both worry time and problem solving.

#### Module: Relapse Prevention

This module is based on a relapse prevention protocol for LICBT [54,68] and is completed at the end of the 12-week intervention period or before if a collaborative decision is made between the parent and the e-therapist. Parents identify warning signs that may indicate relapse using the Five Areas CBT model [88,89] completed in the introduction and psychoeducation module. Next, parents identify what activities, skills, and techniques they have learned and found helpful during EJDeR to inform a staying-well toolkit. Parents are encouraged to make a written commitment to check-in with themselves, initially on a weekly basis, to consider what warning signs they may be experiencing. If parents find themselves experiencing warning signs, they should use their staying-well toolkit to identify how to address these.

### TIDieR Checklist Item 5: Expertise, Background, and Specific Training Given to Intervention Providers

EJDeR is designed to be guided by e-therapists trained in the competencies required to support LICBT [90]. Within the IAPT program [27], guidance is provided by a psychological well-being practitioner workforce, where practitioners receive 9 months of graduate or postgraduate level training and are not required to have a core health or mental health professional qualification [34]. In Sweden, there is no psychological well-being practitioner workforce. Therefore, e-therapists are intended to be psychology program students, in at least their fourth year of study, including a term of advanced studies in CBT and those who have not yet gained an accredited mental health professional qualification.

A 2-day training program for EJDeR was provided to e-therapists by intervention authors PF (IAPT program LICBT national expert advisor and clinical lead, accredited cognitive behavioral psychotherapist and chartered psychologist) and JW (research psychologist, expert in LICBT, and teacher on educational programs to train mental health professionals using LICBT), a Swedish licensed psychologist, and 2 research assistants (MSc level). Training focuses on developing an understanding of (1) LICBT, (2) BA, (3) worry management, (4) difficulties commonly experienced by parents of children treated for cancer, (5) the structure of EJDeR, (6) support protocols, and (7) using the portal.

e-Therapists receive weekly group clinical supervision via videoconferencing or face-to-face with a licensed psychologist with expertise in the population and internet-administered CBT. On-demand individual supervision with a licensed psychologist is provided, if required.

### TIDieR Checklist Item 6: Modes of Delivery

#### The Portal

The portal [64,65] incorporates security and safety features to ensure sensitive information management, including (1) user log-in via bank ID (a citizen authentication system used in Sweden); (2) access through an encrypted connection using an HTTPS protocol; (3) protection of the webserver via Uppsala University’s secure firewall, allowing only http secure traffic; and (4) storage of study data on a separate database to personal data (eg, the parent’s identity and contact details) with both databases encrypted using 256-bit transparent data encryption. User action logging is enabled via action metadata management to allow user behavior analysis, including (1) log-ins; (2) log-outs; (3) opened modules; (4) section views (eg, the library); (5) opening PDFs; (6) homework entries, (7) multimedia (eg, audio and video) file consumption (including play, pause, and stop); and (8) time-stamp data. Message logging is also enabled, for example, the number of automated reminders sent via SMS text messaging or email, and the number of written messages sent between the e-therapist and the parent within the portal. A number of persuasive system design elements [91,92] are integrated to improve intervention adherence: (1) tunneling (eg, intervention content delivered in a predefined step-wise order to guide users through the intervention); (2) tailoring (eg, intervention content is personalized to user needs, ie, their main presenting mental health difficulty); (3) personalization (eg, reminder messages include the parent’s first name); (4) self-monitoring (eg, mood monitoring via a visual analog scale); (5) rehearsal (eg, exercises are repeated); (6) reminders (eg, automated messages to remind parents to perform specific actions); (7) similarity (eg, use of case vignettes); and (8) liking (eg, use of professional illustrations).

#### e-Therapist Guidance

Guidance is provided to parents by a secure inbuilt videoconferencing system, written messages via the portal, and over the telephone. e-Therapists hold an initial assessment session with the parent informed by existing protocols [68] via videoconferencing or telephone. At the end of the assessment, a decision is made concerning which LICBT technique is best suited to the parent depending on their main difficulty (eg, depression or GAD). Thereafter, e-therapists provide weekly guidance via written messages within the portal, informed by evidence suggesting frequent support is associated with adherence [93]. Weekly written messages are informed by an existing brief check-in support protocol [68] and include (1) reviewing and providing feedback on weekly homework exercises; (2) reinforcement of progress made; (3) normalization of any difficulties encountered; (4) assistance with problem solving difficulties and directing the parent to advice in the EJDeR intervention; (5) setting a plan for the use of EJDeR over the coming week; and (6) encouragement to support continued motivation and engagement. The brief check-in support protocol [68] is informed by the ICBT Therapist Rating Scale [94] and designed to minimize the use of undesirable e-therapist behaviors [95]. e-Therapists may provide at-need written support via the portal if requested and are required to respond to parents within 1 working day. Parents receive a booster session via videoconferencing or telephone halfway through EJDeR to review and assess progress, identify and provide assistance for problem solving any difficulties experienced, and provide continued encouragement and motivation.

### TIDieR Checklist Item 7: Location

e-Therapists were located at Uppsala University, Sweden. EJDeR can be assessed on PCs, smartphones, and tablets.

### TIDieR Checklist Item 8: Timing, Duration, and Intensity

EJDeR is designed to be delivered over 12 weeks. The initial assessment session lasts approximately 45 minutes and the booster session lasts for 30 minutes. e-Therapists are expected to spend 20-30 minutes per parent each week, providing weekly written messages via the portal. Parents are expected to complete the introduction and psychoeducation module and one LICBT intervention module (eg, BA or worry management).

### TIDieR Checklist Item 9: Tailoring the Intervention

Content has been closely developed alongside PRPs and has been informed by research identifying the experiences, distress, needs, and preferences for support of parents of children treated for cancer [5,6,49-52]. Examples of tailoring for the population include (1) the use of case vignettes of parents using the intervention, which were informed by our previous research to enhance realism and relevancy [5,51]; (2) professional illustrations depicting parents throughout the intervention; (3) the inclusion of psychoeducation in the context of the situation of being a parent of a child treated for cancer (eg, fear of cancer reoccurrence); (4) the choice between attending the initial assessment session via telephone or videoconference [51]; and (5) the inclusion of a midintervention booster session [51].

### TIDieR Checklist Item 10: Modifications of the Intervention

EJDeR is currently being tested in a single-arm feasibility study, ENGAGE [96,97] (ISRCTN 57233429), with a baseline, posttreatment (12 weeks), and 6-month follow-up, with an embedded qualitative and quantitative process evaluation to inform a future phase III definitive randomized controlled trial. Findings from the embedded qualitative process evaluation will inform future potential modifications to the intervention. Any intervention modifications during the course of the study will be reported in the ENGAGE study results.

### TIDieR Checklist Item 11: Assessing Intervention Adherence (Planned)

Videoconference and telephone guidance sessions are audio-recorded with informed consent. Overall, 15% of written communication and 15% of video or telephone communication between parents and e-therapists are reviewed by a member of the research team to assess e-therapist fidelity to the clinical protocol. Parent activity on the portal is logged to examine parent adherence, including the number of log-ins, opened modules, completed in-module and homework exercises via the portal, and the number of written messages via the portal sent to e-therapists.

### TIDieR Checklist Item 12: Assessing Intervention Adherence (Actual)

Actual adherence to EJDeR will be reported in the results of the ongoing single-arm feasibility study ENGAGE [96].

## Discussion

### Principal Findings

The detailed description of EJDeR, in line with the TIDieR checklist, can help facilitate e-therapist fidelity to the EJDeR protocol during the ENGAGE study [98]. Furthermore, if EJDeR is implemented later, clinical delivery will be replicable.

### Limitations and Strengths

Although public involvement was embedded within intervention development and resulted in valuable feedback and intervention changes, involvement was at a consultation level, with feedback provided on materials already developed by the research team. Involvement may have been enhanced by the greater engagement of PRPs earlier in the process. For example, holding in-depth discussion groups, involvement in writing the intervention, and development of case vignettes to add extra authenticity. PRPs only provided feedback on a written version of EJDeR and not when EJDeR was uploaded onto the portal, and was, therefore, reviewed outside of its intended context. However, an important objective of the ongoing study ENGAGE is to examine the acceptability and feasibility of EJDeR in more depth.

EJDeR does not include the collection of routine weekly clinical outcome measurements for clinical purposes, for example, to help inform treatment decisions. Instead, weekly clinical outcome measurements (depression, Patient Health Questionnaire-9; GAD, GAD-7; posttraumatic stress symptoms, PTSD Checklist for *DSM-5*, and PTSD Checklist-Civilian Version) were collected via the portal to inform a process evaluation for research purposes only [96]. Collection of clinical outcome measurements on a session-by-session basis is a core feature of the stepped care model to inform the treatment planning [27] and a core feature of the successful implementation of internet-administered CBT in routine health care [99].

Consistent with the single-strand LICBT interventions developed in England as part of the IAPT program, EJDeR was adapted to enhance the acceptability for the Swedish population. Adopting a more structured framework to inform the cultural adaptation of evidence-based psychological interventions may improve acceptability and relevance [100]. Finally, to ensure consistency with the LICBT approach, EJDeR targets depression and GAD. Therefore, EJDeR does not target all mental health difficulties commonly experienced by parents of children treated for cancer, such as PTSD [50]. Future psychological interventions developed for parents of children treated for cancer may target other difficulties.

Notwithstanding these limitations, the development of EJDeR was informed by a series of iterative research studies, including evidence synthesis, conceptualization of distress, participatory action research, and a cross-sectional web-based survey, and therefore, it is strongly grounded in research on the population. Public involvement was embedded within the intervention development process, resulting in invaluable feedback and intervention changes. Development included translation by native Swedish speakers and subsequent back-translation by a professional translation company.

### Comparison With Prior Work

To the best of our knowledge, this is the first LICBT intervention to be described in detail and in accordance with the TIDieR checklist [47]. Although LICBT clinical protocols have been published [68], the TIDieR checklist represents a systematic and structured approach to facilitating detailed intervention descriptions. The provision of a systematic and structured clinical protocol may be of particular importance, given that therapeutic drift [101] in supporting LICBT is commonly reported [102]. In addition, the content of LICBT interventions differs significantly [34,103] and is poorly described [104]. Furthermore, the use of the TIDieR checklist, alongside the application of further intervention fidelity measures, will facilitate determining the extent to which EJDeR is delivered as planned in the ENGAGE study, thereby increasing confidence in the results of any subsequent effectiveness trial [98].

### Conclusions

Informed by phase I (development) of the Medical Research Council guidance for the development and evaluation of complex interventions [46], an overview of the development process is provided, along with a detailed description of the EJDeR intervention informed by the TIDieR checklist. The provision of a detailed and structured intervention protocol is of particular importance for the implementation of evidence-based treatments and reduction of research waste [48], providing procedures to maximize fidelity to protocols [98]. Reducing therapist drift is a core feature associated with the successful implementation of internet-administered LICBT [99].

## Abbreviations

BA: behavioral activation
CBT: cognitive behavioral therapy
EJDeR: internetbaserad självhjälp för föräldrar till barn som avslutat en behandling mot cancer
GAD: generalized anxiety disorder
HICBT: high-intensity cognitive behavioral therapy
IAPT: Improving Access to Psychological Therapies
LICBT: low-intensity cognitive behavioral therapy
PRP: parent research partner
PTSD: posttraumatic stress disorder
SOC: selection, optimization, and compensation
TIDieR: Template for Intervention Description and Replication
U-CARE: Uppsala University Psychosocial Care Programme
